# Supplementary material for: Biofilm-Based Biocatalysis for Galactooligosaccharides Production by the Surface Display of β-Galactosidase in Pichia pastoris
Source: Int J Mol Sci. 2023 Mar 30;24(7):6507. doi: 10.3390/ijms24076507 (PMC10094928; doi:10.3390/ijms24076507)
Supplement: Supplementary file 1 [file ijms-24-06507-s001.zip › ijms-2305483-supplementary.pdf]

**Supplemental Figures**

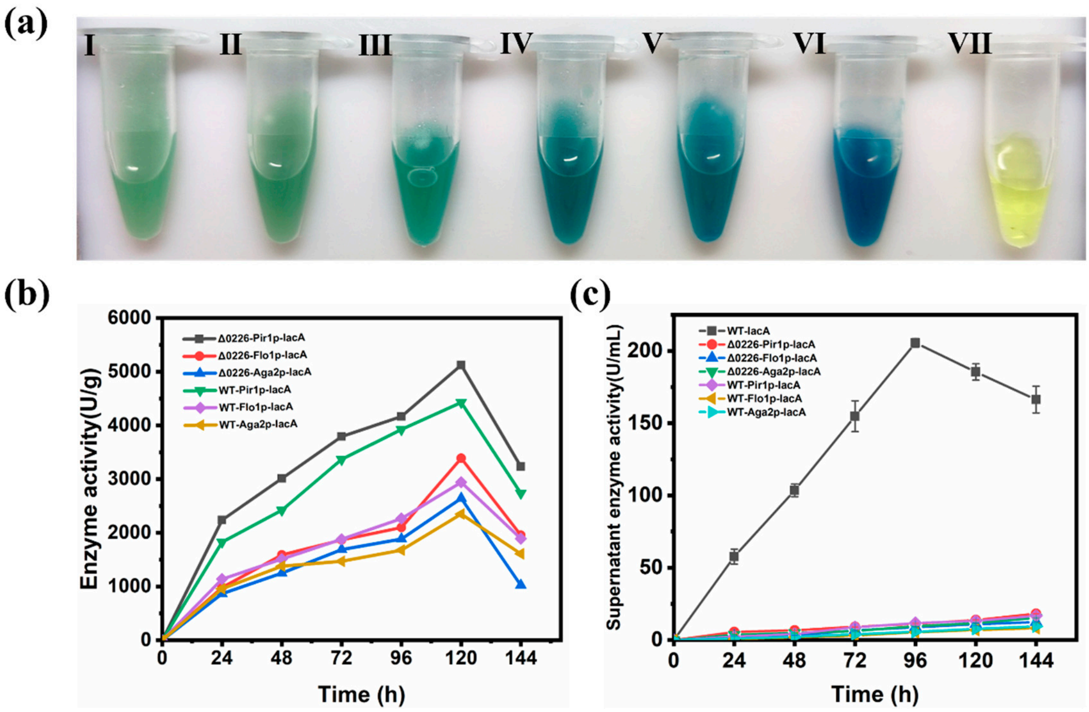


**Figure S1** Expression of enzymes from six surface-displayed engineered strains.

(a) I: WT - Aga2p-lacA, II: Δ0226-Aga2p-lacA, III: WT-Flo1p-lacA, IV: Δ0226-Flo1p-lacA, V: WT-Pir1p-lacA, VI: Δ0226-Pir1p-lacA, VII: control. the darker the color is, the better the expression of β-galactosidase. The enzyme activity of Δ0226-Pir1p-lacA, Δ0226-Flo1p-lacA, Δ0226-Aga2p-lacA, WT-Pir1p-lacA, WT-Flo1p-lacA, and WT-Aga2p-lacA in (b) cell precipitates and (c) supernatant, respectively. The enzymatic activity of Δ0226-Pir1p/ Flo1p / Aga2p -lacA was higher than that of WT-Pir1p/ Flo1p / Aga2p -lacA, indicating that knockout of Δ0226 strain could increase the efficiency of surface display and allow more anchoring proteins to be anchored to the cell wall of *P. pastoris*.
